# Somatic mosaicism for Duchenne dystrophy: Evidence for genetic normalization mitigating muscle symptoms

**DOI:** 10.1002/ajmg.a.32891

**Published:** 2009-06-15

**Authors:** Akanchha Kesari, Robert Neel, Lynne Wagoner, Brennan Harmon, Christopher Spurney, Eric P Hoffman

**Affiliations:** 1 Research Center for Genetic Medicine, Children's National Medical CenterWashington, District of Columbia; 2 Department of Neurology, University of Cincinnati Medical CenterCincinnati, Ohio; 3 Department of Cardiology, University of Cincinnati Medical CenterCincinnati, Ohio; 4 Department of Cardiology, Children's National Medical CenterWashington, District of Columbia

**Keywords:** genetic normalization, somatic mosaic, dystrophin

## Abstract

We describe a young adult male presenting with cardiac failure necessitating cardiac transplantation 7 months after presentation. Skeletal muscle biopsy showed mosaic immunostaining for dystrophin. DNA studies showed somatic mosaicism for a nonsense mutation in the dystrophin gene (Arg2905X). The frequency of normal versus mutant genes were determined in blood/DNA (50:50), muscle/DNA (80:20) and muscle/mRNA (90:10). These data are consistent with genetic normalization processes that may biochemically rescue skeletal muscle in male somatic mosaic patients mitigating muscle symptoms (gradual loss of dystrophin-negative skeletal muscle tissue replaced by dystrophin-positive stem cells). To our knowledge, this is only the second reported case of a clinically ascertained patient showing somatic mosaicism for Duchenne muscular dystrophy (DMD). We hypothesize that many somatic mosaic males for DMD exist, yet they are not detected clinically due to genetic normalization. Somatic mosaicism for DMD should be considered in acute heart failure with dilated cardiomyopathy, as genetic normalization in heart is unlikely to occur.

## INTRODUCTION

Duchenne muscular dystrophy (DMD) is an X linked recessive disorder that occurs in 1 in 3,500 males in all world populations [Hoffman et al., 1988]. DMD is a progressive proximal muscular dystrophy with early calf hypertrophy, elevations of serum creatine kinase, and dystrophic muscle pathology [Ishpekova et al., 1999]. Dilated cardiomyopathy develops relatively late in the disease process. Patients with less severe Becker muscular dystrophy (BMD) variant can show cardiomyopathy as the presenting symptom. Female carriers also show heart abnormalities upon electrocardiographic analysis; however it is rarely life threatening [Politano et al., 1996; Cox and Kunkel, 1997; Hoogerwaard et al., 1999].

A singular feature of *DMD* is the very high spontaneous mutation rate of the large *DMD* gene (2.4Mb), with 1/10,000 of all germ line cells (sperm, eggs) showing de novo mutations [Caskey et al., 1980; Haldane, 2004]. The high germ-line (meiotic) mutation rate leads to a high proportion of boys with DMD with no previous family history, where the mother is not a carrier of her son's mutation. Typically, high germ line mutation rates predict high somatic (mitotic) mutation rates, leading to cases of somatic mosaicism. For example, in DMD, male embryos may sustain a mutation of the *DMD* gene early in embryonic development, leading to a patient with populations of both abnormal (dystrophin-negative) and normal (dystrophin-positive) in tissues expressing the gene (muscle, heart, smooth muscle). Despite the expected high incidence of somatic mutations in the dystrophin gene we are aware of only three published cases of male somatic mosaics for DMD, two cases ascertained at autopsy [Saito et al., 1995; Uchino et al., 1995; Deburgrave et al., 2007]. The scarcity of published cases of somatic mosaics could reflect a lower mutation rate in somatic cells relative to germ line cells, or alternatively, somatic mosaics may be poorly ascertained due to mild or unexpected clinical symptoms.

In this report we present a case of somatic mosaicism in *DMD* gene presenting with acute cardiac failure. This broadens the clinical phenotypes associated with dystrophinopathy. We also show data consistent with genetic normalization processes previously reported in female carriers, where the muscle becomes increasingly dystrophin-positive (normal) with advancing age [Pegoraro et al., 1995]. We hypothesize that genetic normalization in skeletal muscle obviates muscle symptoms in many or most male somatic mosaics for DMD.

## PATIENT AND METHODS

A 20-year-old African American male sought evaluation after a 2-week history of increasing fatigue, shortness of breath and a syncope. Medical history was significant only for mild intermittent asthma. At initial evaluation, an echocardiogram showed severely depressed left ventricular systolic function. The patient was placed on inotropic support and underwent cardiac catheterization where an intra-aortic balloon pump was placed. Possible muscular weakness and persistently elevated serum creatine kinase prompted a neurological consult that found mild weakness (4/5 MRC) of the deltoids and hip flexors, but was otherwise normal. An EMG was obtained that was myopathic and biopsies of skeletal and cardiac muscle were obtained. The patient was discharged home on a continuous inotrope infusion. His cardiac function did not improve and he underwent cardiac transplantation approximately 7 months later.

### Biochemical and Molecular Analysis

The patient's muscle biopsy was studied by immunostaining for dystrophin, alpha-sarcoglycan and merosin, and immunoblotting for dystrophin and dysferlin (CNMC IRB protocol #2405).

Muscle genomic DNA was isolated from approximately 10mg of muscle biopsy using Genomic DNA Purification Kit by Qiagen (Valencia, CA). Peripheral blood samples were obtained from the patient, and his parents, and genomic DNA was isolated. Muscle biopsy RNA was purified, and converted to cDNA. One hundred nanograms of genomic DNA and biopsy cDNA were used for MLPA reactions and cDNA–MLPA as previously described [Kesari et al., 2008]. Mutation numbering is based on the DMD coding DNA reference sequence GenBank ID NM_004006.1. The case reported here corresponds to subject #70 in Kesari et al. 2008.

### Allele Specific Expression Assays

Applied Biosystem's Taqman® Assays-on-Demand was used for quantitation of normal versus mutant alleles in each sample. The template (both cDNA and the corresponding genomic DNA) was mixed with 900nM forward and reverse PCR primers, 200nM fluorescent allele discrimination probes and TaqMan® Universal PCR Master Mix, No AmpErase® UNG (Applied Biosystems [ABI], Foster City, CA). Five replicates were run for each reaction. The amplification and probe release was done in the ABI 7900 Real-Time PCR System. Fluorescent readings for both the common and rare alleles are taken after each of the 44 amplification cycles. Data analysis for allele expression is done using Ct values, normalizing the cDNA values for each allele to the genomic DNA values for that same allele.

## RESULTS

### Pathological Findings

**Fig. 1:**
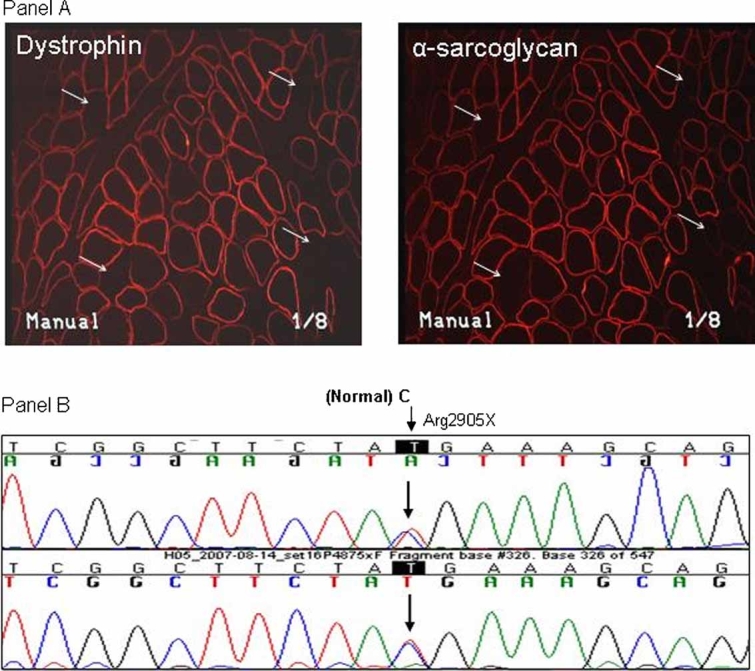
Molecular analysis of a somatic mosaic of DMD. **Panel A**: Shown is immunoflurorescence staining of serial sections of skeletal muscle of the patient for dystrophin (left panel), and alpha-sarcoglycan (right panel). Arrows show dystrophin-negative fibers, and these same fibers show secondary deficiency of alpha-sarcoglycan. **Panel B**: Shown is automated sequence analysis of dystrophin gene exon 59 in cDNA from the patient. Apparent heterozygosity for a C>U change at position 8713 (r.8713c>u) is seen, predicted to result in a stop codon (Arg2905X) in a subset of the patient's genes. Quantitative TaqMan assay data proved that the patient was a somatic mosaic for this mutation.

Right ventricle endomyocardial biopsies showed nonspecific changes of focal myocyte hypertrophy with patchy subendocardial fibrosis, and no significant inflammation. The skeletal muscle showed mild chronic myopathic changes including fiber size variation and increased central myonuclei. Dystrophin immnuostaining showed clear subpopulations of dystrophin-negative and positive myofibers with positive fibers predominating (Fig. 1A; left panel). Serial sections also showed secondary deficiency of α-sarcoglycan in dystrophic negative fibers (Fig. 1A; right panel). Both heart and skeletal muscle tissue tested negative for enteroviruses and influenza B.

Pathological analysis of the explanted heart showed no gross abnormalities of the coronary arteries. There was four chamber dilation and subendocardial fibrosis. H&E stained tissue sections displayed myocyte hypertrophy, subendocardial fibroelastosis and a zone of replacement fibrosis in the left ventricular wall. No frozen tissue of the diseased heart was available for molecular studies.

### Molecular Findings

Genomic DNA and cDNA from muscle biopsy tested negative for deletions and duplications of the 79 exons of the *DMD* gene using MLPA. Complete cDNA sequencing was done, and apparently heterozygosity for a C>T (U) change identified at position 8713 (r.8713c>u), predicted to cause a nonsense mutation (Arg2905X; Fig. 1, Panel B). This mutation was not seen in either parent by MLPA or sequence analyses. A series of known *DMD* gene polymorphisms were identified in the patient that were shared with the mother, and were hemizygous in the patient (Table I), proving somatic mosaicism for the mutation.

**TABLE I:** Biochemical and Molecular Feature of Patient

Age at biopsy	Histopathology	Immunoblot (N=427kDa)	Immunostaining	Change at RNA/protein	Polymorphism and frequent variants
20	Very mild dystrophy	427/100%	Mosaic pattern	r.8713c>u (p.Arg2905X)	r.−8u>a; r.837g>a (p.Thr279Thr) E9; r.7096c>a (p.Gln2366Lys) E48 FV

The reference sequence NM_004006.1 has been used to name the alterations in the sequence, FV—frequent variable as reported in the Leiden database.

Table also shows the point mutation (heterozygous) and polymorphisms and frequent variants (hemizygous) detected in the patient by cDNA sequencing.

**Fig. 2:**
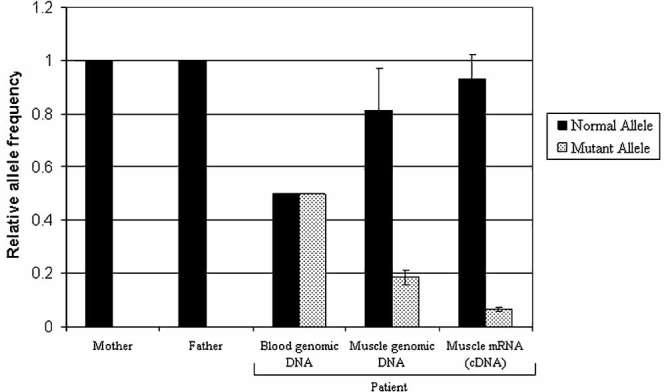
Quantitative allele discrimination assays suggests genetic normalization in muscle. Shown is the relative percentage of normal versus mutant (Arg2905X) genes in the patient's parents, and the patient (peripheral blood DNA, muscle DNA, muscle RNA [cDNA]). Peripheral blood DNA shows equal proportions of normal versus mutant genes (50:50), whereas muscle genomic DNA shows a reduction in mutant genes to 20%, and muscle RNA to 10%. These data are consistent with genetic normalization causing the muscle to become increasingly dystrophin-positive with advancing age.

To determine if genetic normalization had occurred in the male somatic mosaic here, we tested the ratio of normal versus mutant genes in peripheral blood, muscle genomic DNA, and muscle RNA (cDNA; Fig. 2). Muscle showed a much lower proportion of mutant genes, in both DNA (20%) and cDNA (10%). This result is consistent with genetic normalization of muscle, as previously shown for female carriers, and may explain the mild muscle pathology and biochemical findings.

## DISCUSSION

We describe a young male adult presenting with cardiac failure followed by cardiac transplantation. Evaluation for neuromuscular disease (dystrophy, myositis), resulted in the discovery of mosaic immunostaining for dystrophin. Subsequent molecular analysis showed somatic mosaicism for a nonsense mutation in the dystrophin gene (Arg2905X). To our knowledge, this is the first report of DMD somatic mosaicism in a living patient.

The young man's presentation was most consistent with acutely decompensated heart failure secondary to muscular dystrophy associated dilated cardiomyopathy. A similar presentation could be expected with viral myocarditis, but the patient had no significant viral symptoms or fever and serum viral studies for coxsackie B, CMV, HIV, Influenza A and B, Hep B were all negative. PCR viral studies of the skeletal muscle biopsy were negative for enteroviruses and influenza B. The cardiac histological findings, approximately 2 weeks after presentation, do not meet the Dallas criteria for diagnosis of myocarditis [Aretz et al., 1987]. The presentation is also less consistent with ischemic heart disease since there were no significant ST-T wave changes on his presenting electrocardiogram, no regional wall motion abnormalities on echocardiogram and the coronaries were normal on evaluation of the explanted heart.

The patient had a mixture of normal and mutant genes in peripheral blood and skeletal muscle, however the clinical presentation was in heart. In the patient's blood, we found the ratio of normal/mutant to be 50:50, functionally the same as most female carriers (due to random X inactivation) [Pegoraro et al., 1995]. Cardiac muscle shows only two or so per cardiocyte, and thus the somatic mosaic male patient, like female carriers, would be expected to have populations of both dystrophin-positive and dystrophin-negative cardiocytes. Cardiocytes are less affected by dystrophin-deficiency than myofibers, however cardiocytes are generally incapable of regeneration leading to a late-stage cardiomyopathy. We suspect that the acute cardiac failure seen in the somatic mosaic patient presented here is a combination of the failure of cardiac muscle to regenerate, and a disproportionate number of dystrophin-negative cells comprising his heart. This interpretation is consistent with a reported high incidence of heart abnormalities in female carriers. In our previous study of 46 female manifesting carriers, 5 of 7 ECGs were reported as abnormal, and 2 patients presented with cardiac symptoms [Hoffman et al., 1992]. Similar data have been found in additional reports [Kamakura et al., 1990; Mirabella et al., 1993; Kinoshita et al., 1995; Politano et al., 1996]. We cannot rule out that male sex and exercise did not exacerbate the cardiac symptoms as well.

Of note, we found that the patient's muscle tissue showed a much lower relative amount of mutant genes (20%) compared to peripheral blood (50%). There are two possible explanations for this discordance between muscle and blood. The first is that he may by chance have segregated more dystrophin-positive stem cells into the region of muscle biopsies. An alternative explanation is the somatic loss (necrosis) of dystrophin-negative muscle fibers with subsequent regeneration by dystrophin-positive stem cells. This process has been demonstrated in the majority of manifesting female carriers (functionally somatic mosaics, but due to X inactivation rather than somatic mutation) [Pegoraro et al., 1995]. This previous study found that 11/14 of clinically manifesting carriers (80%) showed an average of threefold increase in muscle dystrophin-positive nuclei compared to blood nuclei, strongly arguing for replacement of dystrophin-negative myofibers with dystrophin positive skeletal muscle over time (genetic normalization). This process explains the reduction in serum creatine kinase in female carriers with advancing age, and the age-related improvement in clinical symptoms that can be seen with some manifesting carriers [Pegoraro et al., 1995]. Heart tissue is unable to regenerate, and thus heart is incapable of showing genetic normalization (other than loss of dystrophin-negative regions to fibrosis). Nonsense-mediated decay is a common mechanism by which mRNA levels that contain premature stop mutations are reduced in tissue. This seems a likely explanation for the lower levels of mutant mRNA in skeletal muscle (10%) compared to the proportion in genomic DNA (20%).

Our data suggesting genetic normalization in skeletal muscle in this somatic mosaic patient may explain the paucity of male somatic mosaics for DMD, despite the expected high incidence of such cases based on mutation rates. The genetic normalization process, where dystrophin-negative muscle is gradually replaced by dystrophin-positive muscle as a function of age, would be expected to preclude the onset of skeletal muscular symptoms (weakness). This is in contrast to the heart, where the lack of regeneration in the heart prevents genetic normalization from occurring, and then leading to preferential expression of cardiac symptoms. This case suggests that male somatic mosaics for DMD may present with cardiomyopathy, not skeletal muscle symptoms, and that this diagnosis should be considered in idiopathic cardiomyopathy. Possible screening tests for identifying male somatic mosaics for DMD could include a persistence of the muscle (MM) isoforms of creatine kinase (reflective of a subclinical muscular dystrophy).
